# Glutathione affects the transport activity of *Rhizobium leguminosarum* 3841 and is essential for efficient nodulation

**DOI:** 10.1093/femsle/fnx045

**Published:** 2017-02-23

**Authors:** Guojun Cheng, Ramakrishnan Karunakaran, Alison K. East, Olaya Munoz-Azcarate, Philip S. Poole

**Affiliations:** 1 College of Life Science, South-Central University for Nationalities, Wuhan 430074, China; 2 Department of Molecular Microbiology, John Innes Centre, Norwich Research Park, Norwich, NR4 7UH, UK; 3 Department of Plant Sciences, University of Oxford, South Parks Road Oxford, OX1 3RB, UK

**Keywords:** *Rhizobium*, glutathione, nitrogen fixation, pea, nodulation

## Abstract

As glutathione (GSH) plays an essential role in growth and symbiotic capacity of rhizobia, a glutathione synthetase (*gshB*) mutant of *Rhizobium leguminosarum* biovar *viciae* 3841 (Rlv3841) was characterised. It fails to efficiently utilise various compounds as a sole carbon source, including glucose, succinate, glutamine and histidine, and shows 60%–69% reduction in uptake rates of glucose, succinate and the non-metabolisable substrate α-amino isobutyric acid. The defect in glucose uptake can be overcome by addition of exogenous GSH, indicating GSH, but not its bacterial synthesis, is required for efficient transport. GSH is not involved in the regulation of the activity of Rlv3841's transporters via the global regulator of transport, Pts^NTR^. Although lack of GSH reduces transcription of the branched amino acid transporter, this was not the case for all uptake transport systems, for example, the amino acid permease. This suggests GSH alters activity and/or assembly of transport systems by an unknown mechanism. In interaction with plants, the *gshB* mutant is not only severely impaired in rhizosphere colonisation, but also shows a 50% reduction in dry weight of plants and nitrogen-fixation ability. This reveals that changes in GSH metabolism affect the bacterial–plant interactions required for symbiosis.

## Teaser

Glutathione is needed for bacteria to fix nitrogen efficiently in symbiosis with legumes.

## INTRODUCTION

Rhizobia are gram-negative bacteria that are able to establish an effective nitrogen-fixing symbiosis with their plant hosts (Oldroyd *et al.*^2011^; Udvardi and Poole 2013). A host plant produces a specific chemical signal molecule (flavonoid) that is perceived by the bacterium, and in response rhizobia produce lipo-chitooligosaccharides (Nod factors) leading to root hair curling which surrounds the bacteria (Long 1996). Following endocytosis into an infected cortical cell, rhizobia are engulfed by a plant-derived membrane (symbiosome membrane), to form a symbiosome (Clarke *et al.*^2015^). Inside symbiosomes, rhizobia differentiate into nitrogen-fixing bacteroids. During the infection process and nodule formation, rhizobia are greatly dependent on glutathione (GSH) to protect themselves against oxidant damage (Harrison *et al.*^2005^). The thiol tripeptide GSH (L-glutamyl-L-cysteinylglycine) is abundant in living cells where it acts as an antioxidant either directly, by interacting with reactive oxygen and nitrogen species (ROS and RNS, respectively) and electrophiles, or by operating as a cofactor for various enzymes (Ribeiro *et al.*^2015^). As well as being synthesised within the bacteria, GSH is produced by plant roots during the process of nodule formation (Becana *et al.*^2010^; Matamoros *et al.*^2015^). GSH biosynthesis in bacteria is a two-step process catalysed by ATP-dependent enzymes. Glutamic acid and cysteine are conjugated by γ-glutamylcysteine synthetase (encoded by *gshA*), and its product, γ-glutamylcysteine, is linked to glycine to form GSH in a reaction catalysed by glutathione synthetase (encoded by *gshB*).

In addition to its antioxidant role in nodule formation, it has been shown that free-living rhizobia, such as *Rhizobium etli, R. tropici, Ensifer (Sinorhizobium) meliloti* and *Bradyrizobium* sp. require GSH to combat environmental stress and to efficiently utilise various compounds, such as glucose, succinate, glutamine, alanine and histidine, as their sole carbon source (Riccillo *et al.*^2000^; Muglia *et al.*^2008^). In *R. etli*, GSH is required for glutamine utilisation and symbiotic effectiveness (Taté *et al.*^2012^).

Rhizobia are known to be especially rich in ATP-binding cassette (ABC) transporters (Walshaw and Poole 1996). In *R. leguminosarum* biovar *viciae* 3841 (Rlv3841), amino acid transport occurs predominantly via two ABC transport systems, termed the general amino acid permease (Aap) and the branched-chain amino acid permease (Bra) (Hosie *et al.*^2002^). Glucose is transported into Rlv3841 cells by at least three different ABC transport systems (Untiet *et al.*^2013^). In *R. leguminosarum*, transport of L-malate, fumarate and succinate occurs via the C4-dicarboxylic transport (Dct) system (Reid and Poole 1998). It is worth noting that Pts^NTR^ acts as a global regulator of many functions in the cell, including regulating ABC transport systems at the post-transcriptional level. The first component of this system, PtsP, is phosphorylated by PEP and transfers this phosphate to PtsN1 and PtsN2 via a small protein Npr/Hpr. Inactivation of PtsP, Npr or both copies of PtsN results in a severe inhibition of transport of amino acids by Aap and Bra (Prell *et al.*^2012^; Untiet *et al.*^2013^). The precise mechanism by which PtsN regulates ABC transporters is unknown, but the overlap with the phenotype of strains altered in GSH metabolism indicated the possibility that Pts^NTR^ might control ABC transporters via GSH levels.

Given the importance of GSH in many rhizobia during symbiosis, we consider it important to understand the effects of GSH on carbon compound transport and metabolism in both free-living and symbiotic *R. leguminosarum*.

## MATERIALS AND METHODS

### Bacterial growth and media

Strains, plasmids and primers used in this study are listed in Table 1. *Rhizobium leguminosarum* strains were grown at 28°C in either Tryptone Yeast extract (TY) (Beringer 1974) or acid minimal salts medium (AMS) (Poole *et al.*^1994^) with 10-mM D-glucose (Glc) or 10-mM di-sodium succinate (Suc) as carbon sources and 10-mM NH_4_Cl as a nitrogen source, unless otherwise described. They were grown on AMS agar containing 5-mM glutamine (Gln), glutamate (Glu), histidine (His), alanine (Ala) or asparagine (Asn) as sole carbon and nitrogen source.

**Table 1.** Strains, plasmids and primers.

Strain, plasmid or primer	Description	Reference, source or DNA sequence (5΄-3΄)^a^
Rlv3841	*R. leguminosarum* biovar*viciae*, Str^r^	Johnston and Beringer (1975)
LMB599	Rlv3841 *gshB*::pKGshB, Str^r^, Neo^r^	This study
LMB675	LMB599[pGshB], Str^r^, Neo^r^, Tet^r^	This study
PtsP107	Rlv3841 Tn5::*ptsP*; Str^r^, Neo^r^	Prell *et al.* (2012)
pRK415-1	Plasmid; IncP broad host range cloning vector, Tet^r^	Karunakaran *et al.* (2006)
pK19mob	Plasmid; pUC19-derivative, plasmid,*lacZ mob*; Km^r^	Karunakaran *et al*. (2010)
pRK2013	Helper plasmid for mobilising plasmids; Km^r^	Figurski and Helinski (1979)
pKGshB	Plasmid; pr1373/pr1374 PCR product cloned in pK19mob, Km^r^	This study
pGshB	Plasmid; pr1490/pr1491 PCR product cloned in pRK415-1, Tet^r^	This study
pK19A	Primer; pK19mob plasmid mapping primer	Karunakaran *et al.* (2010)
p1022, p1023	Primers for qRT-PCR of *aapJ*	Mulley *et al.* (2011)
p1028, p1029	Primers for qRT-PCR of *aapQ*	Mulley *et al.* (2011)
p1030, p1031	Primers for qRT-PCR of *braC*	Mulley *et al.* (2011)
p1032, p1033	Primers for qRT-PCR of *braD*	Mulley *et al.* (2011)
p1034, p1035	Primers for qRT-PCR of *braE*	Mulley *et al.* (2011)
p1038, p1039	Primers for qRT-PCR of *braG*	Mulley *et al.* (2011)
pr1373	Sense primer for PCR of amplifying internal fragment of RL0338 (*gshB*)	AAATCTAGACCGGAG CGCGTCGACCTTGC
pr1374	Antisense prime for PCR of amplifying internal fragment of RL0338 (*gshB*)	AAAAAGCTTCGGCCGCCGACATGCATGTT
pr1375	Mapping PCR primer for checking *gshB* mutation	CGAAGGTGAGGTCCATATCA
pr1490	Sense primer for PCR of complete RL0338 (*gshB*) gene	TTGGATCCAACAGCGGCAACCAGGGCAT
pr1491	Antisense primer for PCR of complete RL0338 (*gshB*) gene	TTGGATCCTCAGCCGCGCTTGCGTTCGAT
pr1562	Sense primer for qRT-PCR of *gyrB1*	GGCATCACCAAAAGGGAAAA
pr1563	Antisense primer for qRT-PCR of *gyrB1*	GCGAGGAGAATTTCGGATCA

a Restriction sites in primer sequences are underlined.

To determine a strain's ability to grow on different compounds, the method described by Taté *et al*. (2012) was used with the media described above (TY and supplemented AMS), with and without addition of exogenous 100 μM GSH as described by Taté *et al*. (2012) and photographed after 3 days’ incubation at 28°C. Antibiotics were used at the following concentrations (μg mL^−1^): carbenicillin (Carb), 50; gentamicin (Gm), 20; kanamycin (Km), 20; neomycin (Neo), 80 (for *R. leguminosarum*), or 250 (for *Escherichia coli*); nyastatin (Ny), 50; tetracycline (Tet), 5; spectinomycin (Spec), 100; streptomycin (Str), 500. Optical density (OD_600_) measurements were automatically recorded every 30 min in an Eon Microplate Spectrophotometer (Bio-Tek) and used for calculation of doubling times for each strain.

### Mutants and plasmids

A mutation in *gshB* (RL0338) of Rlv3841 was made by plasmid integration using single crossover. A 500-bp *gshB* fragment was PCR amplified using primers pr1373/pr1374 (Table 1). The fragment was cloned into *Xba*I*/Hind*III sites of pK19mob, resulting in plasmid pKGshB. Plasmid pKGshB was transferred from *E. coli* to Rlv3841 and recombined into the genomic *gshB* region via single crossover to give strain LMB599. Insertion into the *gshB* gene was confirmed by PCR using pr1375 and a pK19mob-specific primer pK19A (Karunakaran *et al.*^2010^).

To complement the *gshB* mutation in LMB599, primers pr1426 and pr1427 were used to amplify the complete *gshB* gene from Rlv3841. The PCR product was digested with *Bam*HI and cloned into *Bam*HI-digested pRK415-1, resulting in plasmid pGshB. Plasmid pGshB was conjugated into LMB599 using pRK2013 as a helper plasmid to provide the transfer genes, as previously described (Figurski and Helinski 1979), to make complemented strain LMB675.

### Transport assays

*Rhizobium leguminosarum* uptake assays were performed with 25-mM (4.625 kBq of ^14^C) solute (Hosie *et al.*^2002^), using cultures grown in AMS with Glc or Suc to an OD_600_ of ∼0.35. When required, an additional 100-μM GSH was added.

### RNA isolation and quantitative RT-PCR analysis

Independent 50-mL cultures of *R. leguminosarum* strains were grown overnight in AMS Glc in 250-mL flasks at 225 r.p.m. to log phase (OD_600_ 0.3–0.4), in triplicate. Cultures (12 mL) were added directly to RNAlater (Ambion) (24 mL) and centrifuged at 11 000 r.p.m., 4°C in a Sorvall SS-34 rotor centrifuge. RNA was treated twice with TURBO DNase using a DNA-free kit (Ambion). cDNA was synthesised using SuperScript II RT and random hexamers (Invitrogen, Carlsbad, California USA). Quantification of cDNA was carried out using the SensiMix SYBR No-ROX kit (Bioline), and real-time amplification of the PCR products was performed using Bio-Rad CFX real-time PCR system. Primers for amplification by qRT-PCR are detailed in Table 1. PCR conditions consisted of an initial incubation step for 3 min at 95°C, followed by 35 cycles for 5 s at 95°C, 10 s at 62°C and 5 s at 72°C. The *gyrb1* gene was used as a calibrator gene and results were analysed as previously described Prell *et al.* (2009). Statistical analysis of data sets was performed using REST (Pfaffl, Horgan and Dempfle 2002) on data from three independent biological samples (each with three technical replicates).

### Dry weight analysis and acetylene reduction

*Rhizobium leguminosarum* strains were used to inoculate surface-sterilised pea seeds (*Pisum sativum* cv. Avola) at the time of sowing. For dry weight determination, plants were grown as previously described (Poole *et al.*^1994^) in a growth room (16-h light/8-h dark) in 2-L beakers filled with sterile medium-grade vermiculite, watered with nitrogen-free nutrient solution and harvested at 6 weeks. The shoot was removed from the root and dried at 70°C in a dry-heat incubator for 3 days before being weighed. Acetylene reduction was determined at pea flowering (3 weeks) as previously described (Allaway *et al.*^2000^).

### Rhizosphere colonisation

Surface-sterilised pea seeds were grown as described above, but in autoclaved boiling tubes filled with 25 mL of washed fine-grade vermiculite supplemented with 10 mL of nitrogen-free rooting solution. Inoculation with the following ratios of colony forming units (cfu) of Rlv3841 and LMB599 was performed after 7 days: 1000:0, 0:1000, 1000:1000 and 1000:10000. At 7 days post-inoculation (dpi) (14 days after planting), plants were removed from the vermiculite, shoots were cut-off and 20 mL of sterile phosphate-buffered saline was added to the root of each plant and vortexed in Multi Reax system (Heidolph) for 15 min, speed 10 (Karunakaran *et al.*^2006^). Following serial dilution in sterile phosphate-buffered saline, samples were plated onto TY medium containing Str and Ny (both Rlv3841 and LMB599 will grow) or Str, Ny and Neo (only LMB599 will grow), to give the total number of each strain in the pea rhizosphere (Barr *et al.*^2008^). Agar plates were incubated at 28°C for 3 days, colonies counted and percentage of cells recovered calculated.

## RESULTS

### Rlv3841 *gshB* mutant (LMB599) is defective in growth

A mutation was made in the GSH synthetase gene (*gshB*) of Rlv3841 to give strain LMB599. In liquid cultures of TY or AMS with succinate or glucose as a carbon source, GSH-deficient strain LMB599 has doubling times increased by approximately 2-fold compared to the parental strain Rlv3841 (Table 2). Addition of 100-μM GSH to LMB599 cultures reduces the doubling time in glucose-grown cultures from 12.9 to 5.5 h, to give values similar to those of wild-type Rlv3841 (approx. 5.4 h) (Table 2). This shows that the effects of a mutation in GSH synthesis on bacterial growth are overcome by addition of exogenous GSH.

**Table 2.** Growth of wild-type Rlv3841 and GSH-deficient mutant LMB599.

	Doubling time (h)			
Strain	TY	AMS Suc	AMS Glc	AMS Glc/GSH*
Rlv3841	4.8 ± 0.0^a^	6.8 ± 0.0^a^	5.3 ± 0.1^a^	5.4 ± 0.0^a^
LMB599	8.0 ± 0.4^b^	13.7 ± 0.6^b^	12.9 ± 0.2^b^	5.5 ± 0.1^a^

Strains grown in TY or AMS minimal medium supplemented with glucose (Glc) or succinate (Suc) as the sole carbon source. *100-μM GSH added. Data are averages (±SEM) from three independent cultures. ^a,b^ indicate significant difference (*P* ≤ 0.01).

**Figure 1.**
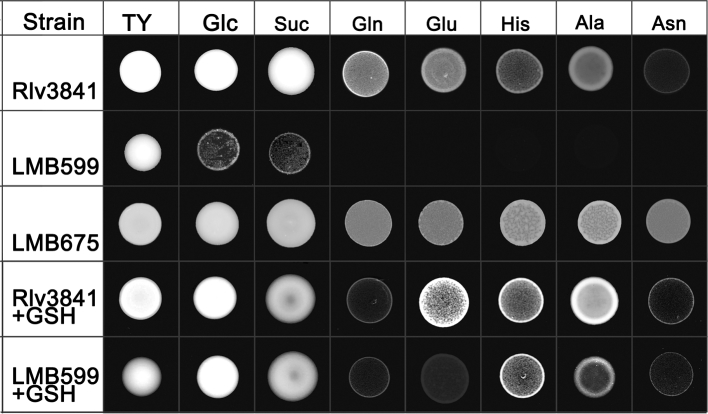
Growth on different media of wild-type Rlv3841, GSH-deficient mutant LMB599 and complemented mutant LMB675. As described by Taté *et al.*(2012), cultures were spotted onto TY agar (complete medium) or AMS agar (minimal medium) containing glucose (Glc) or succinate (Suc) as sole carbon source; glutamine (Gln), glutamate (Glu), histidine (His), alanine (Ala) or asparagine (Asn) as sole carbon and nitrogen sources. + GSH, growth with 100-μM glutathione added exogenously. Plates were incubated at 28°C for 3 days.

On solid media, LMB599 grows poorly on AMS with glucose and succinate as a sole carbon source, while growth is undetectable with amino acids glutamine, glutamate, histidine, alanine and asparagine as sole sources of carbon and nitrogen (Fig. 1). Growth of LMB599 on all the media assayed is restored to wild-type Rlv3841 levels by complementation with a cloned full-length copy of *gshB* carried on a plasmid (strain LMB675; LMB599[pGshB]) (Fig. 1). Indeed, in LMB675 growth on histidine, alanine and asparagine appears to be better than in wild-type Rlv3841. Addition of 100-μM GSH also restores the growth of LMB599 on glucose and succinate, although exogenous GSH has a slightly less clear-cut effect on amino acid transport. In the *gstB* mutant, slight growth is seen on histidine and alanine while there is little discernible growth on glutamine, glutamate and asparagine as sole sources of carbon and nitrogen. Addition of exogenous GSH also adversely affects the growth of Rlv3841on glutamine a sole carbon and nitrogen source (Fig. 1).

### GSH is required by a broad range of carbon transport systems

**Figure 2.**
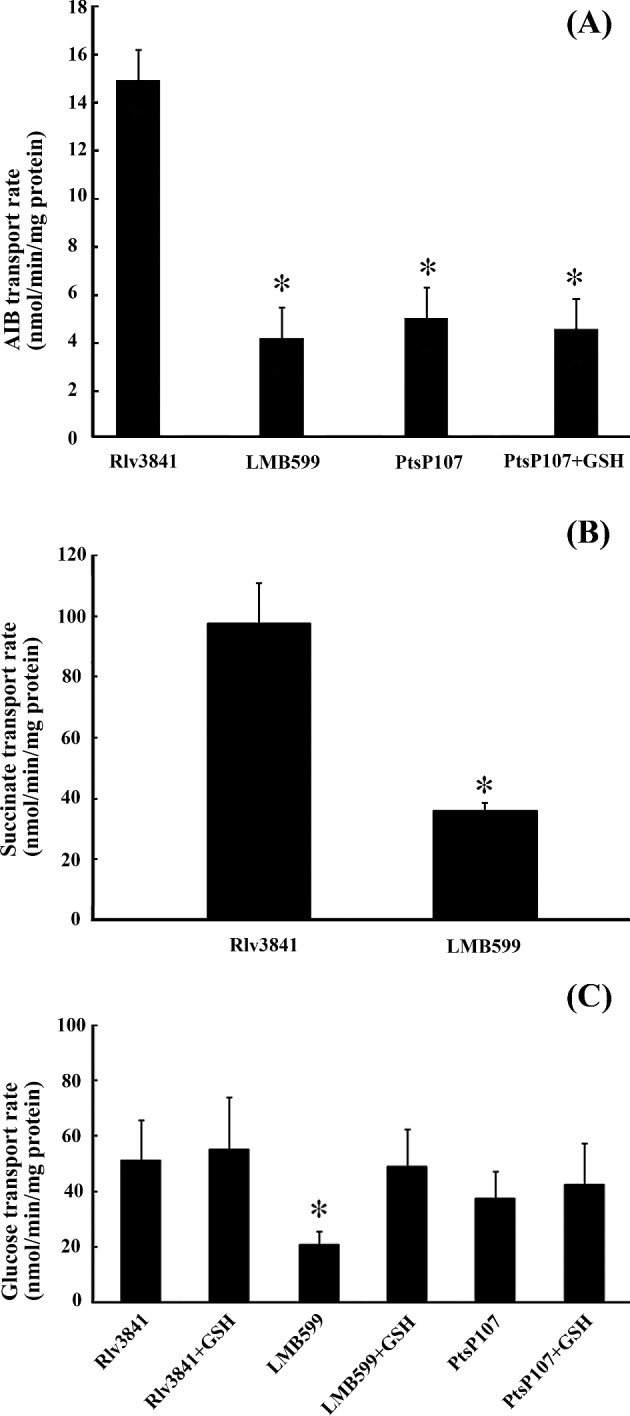
Uptake of AIB, succinate and glucose by strains of *R. leguminosarum*. (**A**) ^14^C-AIB uptake by wild-type Rlv3841, GSH-deficient mutant LMB599 and PtsP107 (*ptsP* mutant) following overnight growth in AMS Glc, with or without addition of 100-μM GSH. An asterisk indicates a significant difference compared to wild-type Rlv3841 (*P* ≤ 0.001). (**B**) ^14^C-Suc uptake by Rlv3841 and LMB599 following overnight growth in AMS Suc. An asterisk indicates a significant difference compared to wild-type Rlv3841 (*P* ≤ 0.05). (**C**) ^14^C-Glc uptake by Rlv3841, LMB599 and PtsP107 following overnight growth in AMS Glc, with or without addition of 100-μM GSH. An asterisk indicates a significant difference compared to wild-type Rlv3841 (*P* ≤ 0.05). All data are averages (±SEM) from ≥three independent cultures.

As LMB599 grows poorly on a range of amino acids, we tested amino acid uptake in LMB599 and compared it with that of wild-type Rlv3841*.* Amino acid transport was measured with the non-metabolisable amino acid α-amino isobutyric acid (AIB) because, like glutamine, it is transported exclusively by the Aap and Bra uptake systems (Hosie *et al.*^2002^). As AIB cannot be metabolised, the measurement of AIB transport is not complicated by secondary metabolic effects. Strain LMB599 shows a 69% reduction in the rate of AIB uptake (Fig. 2A).

As growth on both glucose and succinate as a sole carbon source is also substantially reduced in strain LMB599, it may be that the effects of GSH deficiency are more general than just those observed on amino acid uptake. Uptake of succinate and glucose was measured in strain LMB599 and are reduced by 63% and 60%, respectively (Fig. 2B and C). Addition of 100-μM GSH rescues the defect in glucose transport (Fig. 2C). Thus, in comparison with Rlv3841, LMB599 shows a significant reduction in transport of AIB, succinate and glucose (Fig. 2).

### Role of GSH in altering activity of ABC transport systems

PtsP has been shown to be essential for activation of a broad range of ABC transport systems (Prell *et al.*^2012^), and it is possible that this occurs via a lack of GSH. To test this hypothesis, we examined whether addition of exogenous GSH has any effect on the transport of amino acids and glucose in a *ptsP* mutant, strain PtsP107 (Prell *et al.*^2012^). The significantly lower rate of AIB transport in strain PtsP107 compared to Rlv3841 is not affected by the addition of 100-μM GSH (Fig. 2A). Glucose transport is not significantly different in strains PtsP107 and Rlv3841, and is unaffected by addition of exogenous GSH (Fig. 2C). GSH is found to have no role in the post-translational PTS^Ntr^ system-mediated effects on ABC transport systems.

To investigate whether transcriptional control is involved, qRT-PCR was used to determine whether the Aap and Bra transport systems, i.e. those transporting AIB, are transcriptionally affected in the GSH-deficient mutant. In LMB599, expression of the Bra transporter genes is downregulated significantly (>2-fold, *P* ≤ 0.05) (*braC* 2.97±0.55↓*, braD* 25.24±7.88↓*, braE* 3.85±1.11↓ *and braG* 4.95±2.41↓)**, while expression of genes encoding the Aap permease is not significantly (*P* > 0.05) slightly upregulated (*aapJ* 2.41±0.33↑) or not altered (<2-fold) (*aapQ* 1.36±0.36↑) (where ↓ shows fold decrease in relative expression and ↑ shows fold increase in relative expression compared to Rlv3841). GSH significantly downregulates the transcription of genes encoding the Bra transporter but not those of the Aap transporter which essentially show unchanged expression.

### Nodulation and competitiveness in the rhizosphere of a GSH-deficient mutant

In order to assess the nodulation and nitrogen-fixing capacity of GSH-deficient mutant LMB599, *Pisum sativum* seedlings were inoculated with *R. leguminosarum* strains and the number of nodules per plant, dry weight of plants and acetylene reduction activity measured. Strain LMB599 forms a similar number of nodules as wild-type Rlv3841 (approx. 180); however, there are almost twice as many small (pink and white) nodules (approx. 75% of 177 ± 58) on LMB599-inoculated plants than for those inoculated with wild-type Rlv3841 (approx. 45% of 176 ± 23) (Table 3). The *gshB* mutant LMB599 shows a 50% drop in the dry weight of plants and a 50% decrease in acetylene reduction activity (Table 3). Plants nodulated with strain LMB675, in which the mutation in *gshB* is complemented by a full-length *gshB* gene cloned on a plasmid, have wild-type properties; approximately the same number of nodules per plant, same percentage of small nodules (approx. 49% of 174 ± 22) and reduce acetylene at the same rate as Rlv3841-inoculated plants (Table 3).

**Table 3.** Symbiotic behaviour of wild-type Rlv3841, GSH-deficient mutant LMB599 and complemented mutant LMB675.

	Nodules	Small nodules/total	Acetylene reduction	Dry weight
Strain	per plant	nodules (%)*	(μmoles acetylene per plant per h)	per plant (g)
Rlv3841	176 ± 23^a^	45 ± 5^a^	4.10 ± 0.10^a^	2.38 ± 0.31^a^
LMB599	177 ± 58^a^	78 ± 6^b^	2.01 ± 0.29^b^	1.15 ± 0.27^b^
LMB675	174 ± 22^a^	49 ± 4^a^	3.70 ± 0.19^a^	ND
WC	0	ND	0	0.33 ± 0.13^c^

All data are averages (±SEM) from five, or from ten (dry weight) independent plants. *Small nodules are of length <2 mm and include both pink and white nodules. ^a,b,c^Student's *t* test was performed within each experiment and letters indicate significant difference (*P* ≤ 0.01). WC, water control is uninoculated; ND not determined.

**Figure 3.**
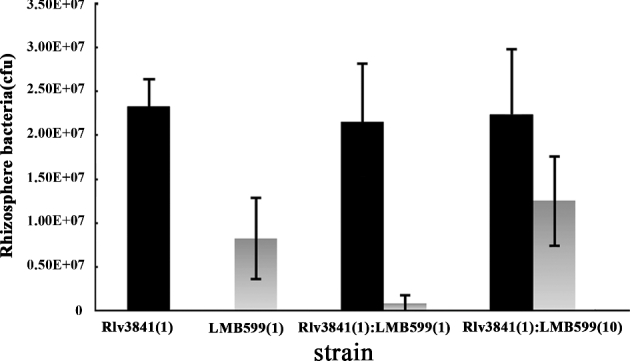
Colonisation of the rhizosphere of pea plants by wild-type Rlv3841 (black) and GSH-deficient mutant LMB599 (grey), inoculated individually and together. Inoculation ratios are given on the *x-*axis, with 1 corresponding to 10^3^ cfu. Rhizosphere bacteria is the average number of bacteria (cfu) recovered per plant (±SEM). Number of replicates = 10.

To determine survival of the GSH-deficient mutant in the pea rhizosphere, a low number of bacteria (10^3^ cfu) were introduced onto seedling roots and the total bacteria determined after 7 days’ plant growth. When individual LMB599 and wild-type Rlv3841 strains were used to inoculate a sterile pea rhizosphere, the population density of the mutant strain recovered is only 35% that of wild type (Fig. 3). Competition between the GSH-deficient mutant and wild type for growth in the pea rhizosphere was measured by inoculating pea roots with both bacterial strains together. When inoculated in equal ratios, LMB599 accounts for only 3% of bacteria recovered (*t* test; *P* ≤ 0.001). Even when strain LMB599 was inoculated at a 10-fold excess over wild type, it accounts for only 36% of the total bacteria recovered (Fig. 3).

## DISCUSSION

In *Rhizobium etli*, the presence of *gshB*, encoding GSH synthetase, is essential for growth on glutamine as the sole source of carbon and nitrogen (Taté *et al.*^2012^). In GSH-deficient strains of this organism, glutamine transport was reported to be the defective step in glutamine utilisation (Taté *et al.*^2012^). Here, we focus on a GSH-deficient strain of *R. leguminosarum* that has an impaired growth phenotype and examine the uptake of various carbon compounds. The *R. leguminosarum gshB* mutant grows poorly on a variety of carbon sources including a sugar (glucose), an organic acid (succinate) and amino acids (glutamine, glutamate, histidine, alanine and asparagine). The growth defect is rescued either with a plasmid encoding a functional *gshB* or, on glucose and succinate and, to some extent, histidine and alanine, by addition of exogenous GSH. GSH, either produced inside the bacterial cell or added externally, affects uptake and/or utilisation of these compounds in *R. leguminosarum*.

In the GSH-deficient mutant, transport assays were used to determine the rate of uptake of three compounds; AIB (an amino acid analogue), glucose and succinate. Since the rate of uptake of non-metabolisable AIB is reduced to a similar extent to those of glucose and succinate (60%–69%), GSH may be required for the full activity of many different transport systems. It is noteworthy that rhizobia have a high number of ABC transport systems including (but not exhaustively) three systems for uptake of glucose (Untiet *et al.*^2013^) and Aap/Bra for amino acids (including AIB) (Hosie *et al.*^2002^).

Since the uptake of many of the carbon and nitrogen sources examined is dependent on ABC transport systems, we thought it possible that the PTS^NTR^ system, which is a global regulator of many transporters particularly ABC transport systems (Prell *et al.*^2012^; Untiet *et al.*^2013^), may exert its effect via GSH. Strain PtsP107 shows significantly reduced transport of AIB; however, exogenously added GSH is unable to rescue this deficiency in transport. This indicates that GSH does not regulate amino acid uptake in the PTS^NTR^ system, and there must be another mechanism(s) affecting transport.

*Rhizobium leguminosarum* has two general amino acid permeases, Aap and Bra, which are both broad specificity amino acid transport systems (Hosie *et al.*^2002^) and able to transport AIB (assayed here) and, more importantly, in the context of testing it is important in the *R. leguminosarum* system, glutamine. We determined whether transcription of these two-transport systems is regulated by GSH. In the *R. leguminosarum gshB* mutant, expression of all Bra system genes tested (*braC*, *braD*, *braE and braG*) is >2-fold downregulated compared to wild-type; however, the expression of Aap system genes *aapJ* and *aapQ* is essentially unchanged. This shows that GSH positively regulates the transcription of *bra* but not *aap* genes. Interestingly, the converse was observed in *R. etli*, where the essential role of GSH in glutamine uptake was through regulation of the Aap transport system and the Bra system was found to be dispensable (Taté *et al.*^2012^). Thus, lack of GSH reduces transcription of some, but not all, *R. leguminosarum* transport operons suggesting that GSH does not globally alter transcription of transport genes by means of a single mechanism. The absence of a general transcriptional effect suggests that GSH may directly or indirectly, for example, via redox changes, alter the assembly or activity of a broad range of transport systems by a combination, or even an unknown, mechanism(s). Lack of a single mechanism may be the reason for the different patterns of growth seen on amino acids in both wild-type and mutant strains when exogenous GSH is added (Fig. 1). The fact that only on glutamine is the growth of wild-type Rlv3841 poorer in the presence of additional GSH than in its absence, suggests that glutamine transport and/or metabolism is adversely affected by excess GSH. Additional GSH restores growth to the mutant only on glutamate, histidine and alanine (not glutamine), while the plasmid-complemented mutant LMB675 grows even better than wild-type on asparagine. Perhaps in this last case there is increased transport of asparagine, possibly due to increased level of GSH (on a multicopy plasmid expression of *gstB* is likely to be higher than from a single chromosomal copy), leading to increased expression of the Bra transporter.

In symbiosis, a *R. leguminosarum* GSH-deficient mutant shows an approx. 50% reduction in the dry weight of nodulated plants and diminished nitrogen-fixing capacity (reduced to approx. 50%), although the total number of nodules was unchanged. During nodule formation and symbiosis, legume roots produce antioxidants including GSH (Becana *et al.*^2010^; Matamoros *et al.*^2015^). As growth defects in the free-living *gshB* mutant are rescued by exogenous GSH, it might have been expected that GSH from the plant would be able to perform the same role in symbiotic interactions. However, any antioxidants, including GSH, produced by pea roots are clearly unable to rescue the *gshB* mutation in symbiosis under these conditions as a significant feature of our study is the large the proportion (approx. 75%) of undeveloped and ineffective nodules formed by the mutant strain. This may be due to early senescence, which was observed in the nodules from *gshB* mutants of *R. tropici*, *Ensifer meliloti* and *R. etli* (Harrison *et al.*^2005^; Muglia *et al.*^2008^). In *R. tropici* and *E. meliloti gshB* mutants, the total number of nodules formed was found to be similar to that from equivalent wild-type strains (Riccillo *et al.*^2000^; Harrison *et al.*^2005^), which is also the case for *R. leguminosarum* in this study. Moreover, the reduced ability of the *R. leguminosarum gshB* mutant to grow effectively in a sterile rhizosphere and to compete with its wild-type parent in colonising plant roots shows that bacterial GSH production is important for adaption to the microenvironment of host plants.

An inability to effectively colonise pea roots, poor nodulation, reduced dry weight of inoculated plants and diminished nitrogen fixation shows that normal GSH metabolism is essential for the whole symbiotic process in *R. leguminosarum*. These observations, together with the poor competitive ability to nodulate plants shown by GSH-deficient mutants of *E. meliloti* (Harrison *et al.*^2005^) and *R. etli* (Taté *et al.*^2012^), suggest that lack of bacterial GSH production has a profound influence on symbiosis in many different rhizobia.
